# The longitudinal associations between perceived importance of the internet and depressive symptoms among a sample of Chinese adults

**DOI:** 10.3389/fpubh.2023.1167740

**Published:** 2023-06-08

**Authors:** Zhengyu Wu, Jiabo Zhang, Maomin Jiang, Jiawen Zhang, Ye-Wei Xiao

**Affiliations:** ^1^1School of Public Affairs, Xiamen University, Xiamen, China; ^2^School of Literature and Media, Lingnan Normal University, Zhanjiang, China; ^3^School of Education, Silliman University, Dumaguete, Philippines; ^4^Department of Physiology, School of Basic Medical Science, Southwest Medical University, Luzhou, China

**Keywords:** internet cognition, internet behavior, internet use, depressive symptoms, adults

## Abstract

**Objective:**

The aim of this study is to examine the extent to which individuals’ cognitive evaluation of the importance of the Internet is associated with depressive symptoms, and in what ways.

**Methods:**

This study utilized a sample of 4,100 participants from the fourth (2016), fifth (2018), and sixth (2020) waves of the China Family Panel Studies. Structural equation modeling was employed for data analysis.

**Result:**

The findings of this study reveal that individuals’ cognitive evaluation of the importance of the Internet in 2016 was positively linked with the frequency of Internet use and subjective socioeconomic status in 2018. Furthermore, the frequency of Internet use and subjective socioeconomic status in 2018 were found to be negatively associated with depressive symptoms in 2020. These results indicate that the perceived importance of the Internet has an indirect effect on depressive symptoms through the identified pathway.

**Conclusion:**

The present findings contribute to the existing literature by highlighting the importance of individuals’ perceived importance of the internet as a significant factor that influences depressive symptoms. The results suggest that policy makers should take actions to increase public awareness of the importance of the Internet in the digital era, and to ensure equitable access to the internet, thus facilitating convenient internet use and helping individuals adapt to the digital age.

## 1. Introduction

The internet has become an indispensable aspect of our daily lives, and its social significance has been globally recognized. According to the International Telecommunication Union’s (1) report in 2022, as many as 4.1 billion individuals worldwide had access to the internet in 2019, enabling them to access a variety of products and services such as online shopping, entertainment, health management apps, and work-related activities on a computer. However, excessive use of the internet can lead to pathological and problematic internet use, which can adversely affect mental health, including depression (2), as several studies have shown (3–5). Depression is a person who is depressed usually experiences several of the following symptoms: feelings of sadness, hopelessness, reduced energy and vitality, slowness of thought or action, and disturbed sleep or insomnia (6). The World Health Organization (WHO) estimates that 5% of adults globally suffer from depression, making it a leading cause of disability and contributing significantly to the global burden of disease (7). The COVID-19 pandemic has dramatically increased the incidence of clinically significant depressive symptoms worldwide (8), amplifying the concerns of both academia and society about its negative impact on mental health and the strain it puts on healthcare systems.

According to the cognitive theory of emotion, emotions stem from the evaluation of stimuli and are influenced by environmental events, physiological conditions, and cognitive processes. Among these factors, cognition plays a critical role in determining the nature of emotions (9). Lazarus further argues that emotions are a result of the interaction between a person and the environment, as individuals evaluate their perceptions, leading to behavioral and physiological changes. In emotional situations, people constantly evaluate events in relation to themselves to comprehend their significance and select appropriate action responses (10). Consequently, an individual’s evaluation or perception of a thing can affect their emotions and behaviors. A notable example of this theory is the misperception of the internet among adolescents, leading to internet addiction and mood disorders like depression (11–13). The perceived importance of the internet (PII) is a common perception among individuals that the internet has a significant impact on their life or work, and they consider it to be essential. Based on the cognitive theory of emotion, individuals with a positive perception of the internet are likely to exhibit positive emotions and behaviors, which can reduce the risk of depression (14). However, this conclusion requires further validation, and the hypothesis deserves further investigation as it offers a new perspective for future studies on internet and depression, beyond just internet behavior to internet cognition.

Previous studies have primarily focused on the relationship between internet use and depressive symptoms, with more emphasis on internet behavior than internet cognition (15–17). For instance, research has shown that the internet can provide valuable information that transcends time and space barriers (18), and when used appropriately, it can help people manage their depressive symptoms and improve their mental well-being (14, 19). Other studies have highlighted the positive effects of proper internet use, such as reducing loneliness, increasing social capital, and enhancing happiness and life satisfaction (20–23). On the other hand, numerous studies have indicated that pathological or problematic internet use can increase the risk of depression and lead to various occupational, social, psychological, and physiological impairments (24–29). Internet addiction, akin to addiction to drugs or alcohol, can exacerbate an individual’s depression and cause a host of problems in different areas of their life (10, 11, 30, 31). Furthermore, studies have found that the internet’s effects on an individual’s mental health are not limited to direct causes; socio-psychological factors can also indirectly influence their depressive feelings. For example, subjective socioeconomic status (SSS) reflects an individual’s subjective perception of their position in the socioeconomic structure, including their attitudes, behaviors, and confidence in their prospects and perceptions of social phenomena (32–35). Recent research has shown that internet use can enhance people’s social capital, happiness, and life satisfaction, leading to an improvement in their perceived subjective socioeconomic status (36), which, in turn, positively affects their mental health and reduces the risk of depression (37, 38). However, there is still much to learn about the complex relationship between internet use and depression, and further investigation is needed to fully understand the impact of internet cognition on depressive symptoms.

**Figure 1:**
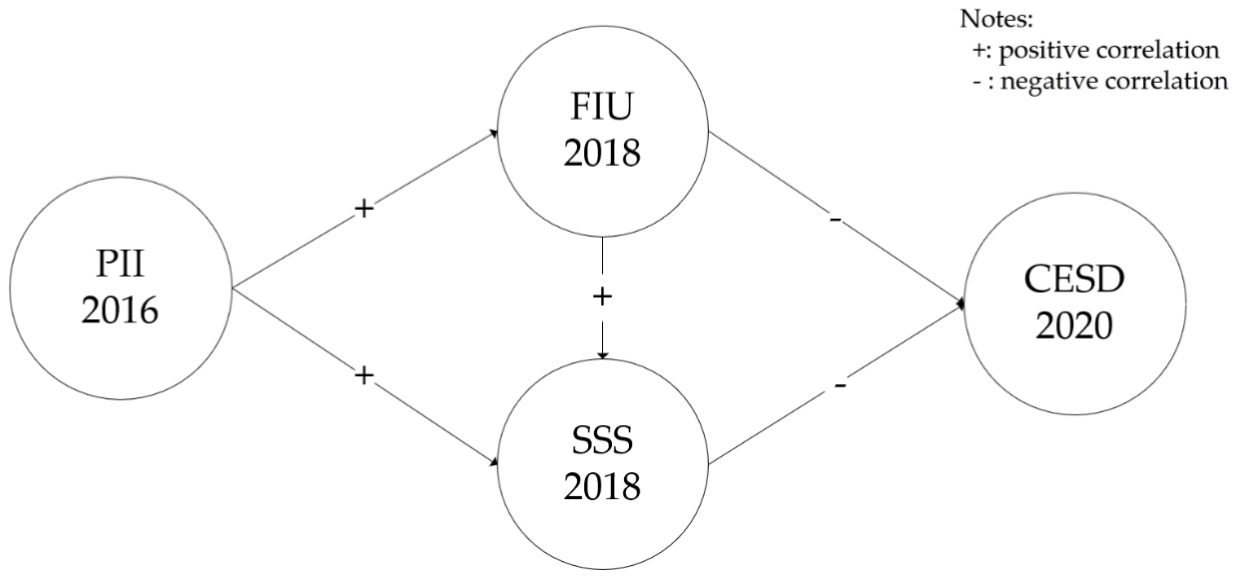
Hypothesized model of the research framework. PII2016, perceived importance of the internet in 2016; FIU2018, The frequency of internet use in 2018; SSS2018, Subjective socioeconomic status in 2018; CESD2020, Self-rated depressive symptoms in 2020.

Taken together, Previous studies have primarily focused on the relationship between Internet behavior and depression, while paying little attention to the role of cognition regarding Internet use (39, 40). Building on the cognitive theory of emotion and existing research, we propose the following hypotheses: (H1) There is a positive relationship between the perceived importance of the Internet and both the frequency of Internet use and subjective socioeconomic status; (H2) The frequency of Internet use and subjective socioeconomic status are negatively associated with depressive symptoms. By investigating the cognitive aspects of Internet use, our study seeks to deepen our understanding of how Internet-related variables contribute to mental health outcomes. In addition, most researchers adopted cross-section data to study internet and depression problems, which could be presumptuous to assume causal relationships. Hence, this study used longitudinal data to investigate mediation associations with an aim to extend the previous conclusions. Hypothesized associations between the variables are presented in Figure 1.

## 2. Methods

### 2.1. Study design and participant

**Figure 2:**
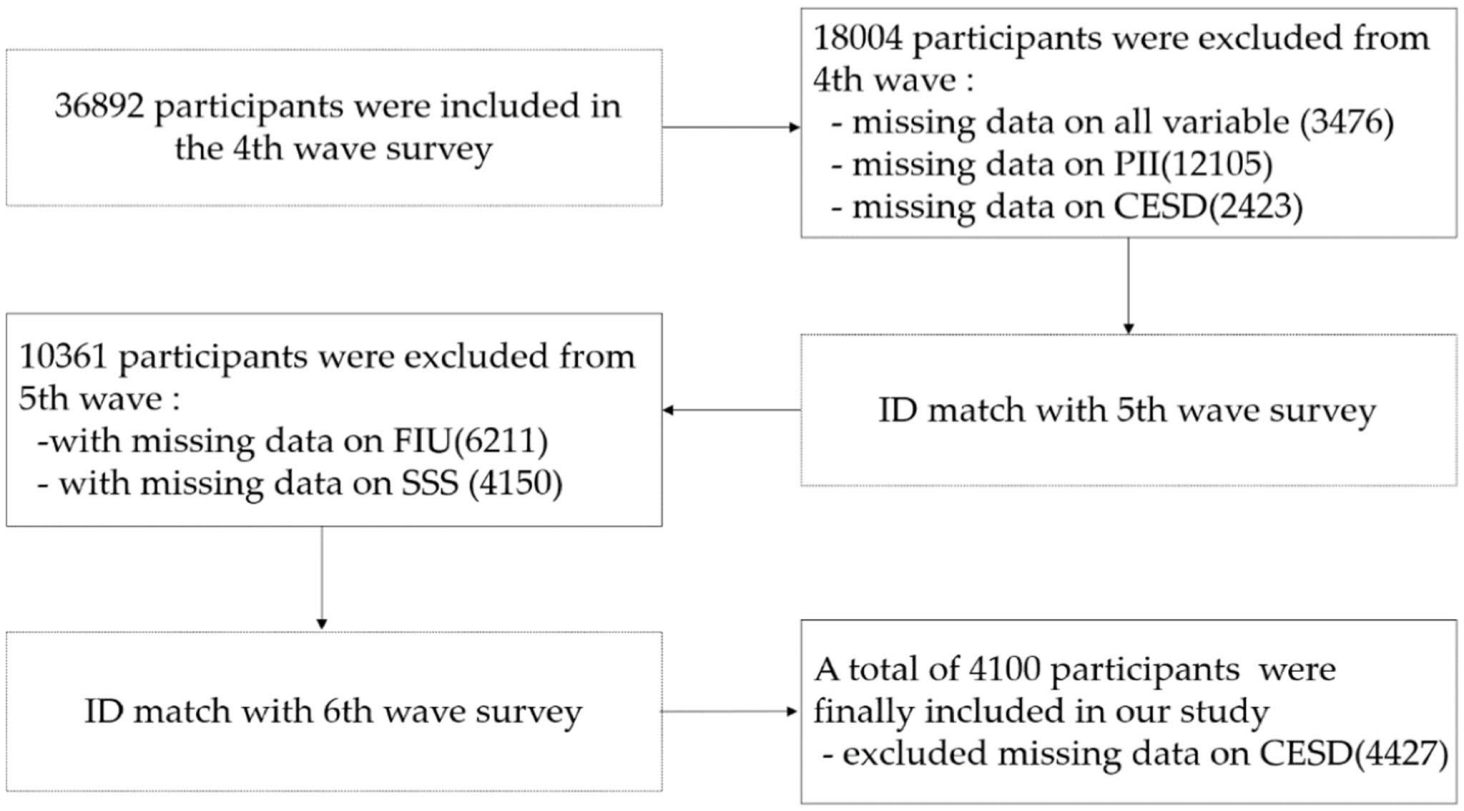
Flow chart depicting participant inclusion and exclusion. PII, perceived importance of the internet; FIU, The frequency of internet use; SSS, Subjective socioeconomic status; CESD, Self-rated depressive symptoms.

The present study utilized data collected from the China Family Panel Studies’ fourth (2016), fifth (2018), and sixth (2020) waves. The China Family Panel Studies (CFPS) is a nationally representative, longitudinal survey administered biannually since 2010 by the Institute of Social Science Survey (ISSS) at Peking University. Its aim is to gather longitudinal data at individual, family, and community levels, primarily focusing on economic activities, educational outcomes, family dynamics and relationships, migration, and health. Data was obtained through face-to-face interviews. The CFPS baseline sample was selected using a multistage probability sampling method with implicit stratification. Each subsample in the CFPS underwent three stages of selection: the county (or equivalent), the village (or equivalent), and the household. Inclusion and exclusion criteria for the current sample were presented in Figure 2 in the form of a flowchart.

### 2.2. Measures

The following items and scales were used in this study:

#### 2.2.1. Perceived importance of the internet (PII)

The perceived importance of the internet (PII) refers to individuals’ belief that the Internet has a significant impact on their life or work, and they consider the Internet to be very important to them. It was measured in this study by four items, including *‘(A1) How important of the internet is for leisure and entertainment? (A2) How important of the internet for daily life? (A3) How important of the internet is for information access? (A4) How important of the internet is to stay in touch with your family and friends?*’ The response options ranged from 1 = very unimportant to 5 = very important. The higher the score, the higher the perceived importance of the internet. The Cronbach’s alpha value of this study was 0.76. The fit indices of the scale are as follows: *X^2^/df* = 4.139, GFI = 0.999, AGFI = 0.995, NFI = 0.998, IFI = 0.999, TLI = 0.992, CFI = 0.999, RMSEA = 0.028, SEMR = 0.006.

#### 2.2.2. Subjective socioeconomic status

Subjective socioeconomic status is more indicative of an individual’s sense of belonging to a particular social class, their confidence in the prospects, perceptions of social phenomena and job opportunities, and their attitudes and behaviors toward themselves and others around them. It was measured in this study with three items, including ‘*(B1) How confident are you about your future? (B2) What is your relative income level in your local area? (B3) What is your social status in your local area?’*. The item was scored on an 5-point Likert scale ranging from 1 = very low to 5 = very high. The higher the score, the higher the sense of subjective socioeconomic status. The Cronbach’s alpha value of this study was 0.63.

#### 2.2.3. The frequency of internet use

The frequency of internet use refers to how often individuals use the Internet for entertainment, life, and work, etc. It was measured in this study with five items, including *‘(C1) In general, how frequently do you use the Internet to socialize? (C2) In general, how frequently do you use the Internet to entertain? (C3) In general, how frequently do you use the Internet to study? (C4) In general, how frequently do you use the Internet to work?*’ The response options ranged from 1 = never to 7 = everyday. The higher the score, the higher the frequency of internet use. The Cronbach’s alpha value of this study was 0.62. The fit indices of the scale are as follows: *X^2^/df* = 10.707, GFI = 0.999, AGFI = 0.984, NFI = 0.992, IFI = 0.993, TLI = 0.931, CFI = 0.993, RMSEA = 0.049, SEMR = 0.011.

#### 2.2.4. Depressive symptoms

Depressive symptoms refer to a set of symptoms associated with a depressive disorder. This study used the 8-item Chinese short version of the Center for Epidemiologic Studies Depression Scale (CES-D) to assess depression symptoms. The CES-D was developed by Radloff (41). Chinese researchers translated and revised the simplified version to create the Chinese version and establish national urban norms for China (42). It was measured in this study using eight items, including *‘(D1) I felt sad, (D2) I felt lonely, (D3) I felt depressed, (D4) My sleep was restless, (D5) I thought my life had been a failure, (D6) I was happy, (D7) I enjoyed life, and(D8) I could not get going.’* Each item was scored on a 4-point Likert scale ranging from 0 = never to 3 = most of the time. The Cronbach’s alpha value of this scale was 0.86. The fit indices of the scale are as follows: *X^2^/df* = 4.885, GFI = 0.996, AGFI = 0.990, NFI = 0.991, IFI = 0.993, TLI = 0.987, CFI = 0.993, RMSEA = 0.031, SEMR = 0.021.

### 2.3. Data analysis

Pearson correlation analysis was used to assess the relationship between the continuous variables AGE, PII, FIU, SSS, and CESD, which were normally distributed. T-tests were performed to compare the mean differences of PII, CESD, and FIU with categorical variables such as gender, education, and marital status. The analysis was conducted using SPSS 26. The measured models for PII, SSS, FIU, and CESD were constructed using observed variables A1-A4, B1-B3, C1-C4, and D1-D8, respectively. These measured models were then used to construct the structural model consisting of PII, SSS, FIU, and CESD. The analysis was performed using AMOS 26. The control variables included age, gender, education length, marital status, and baseline depressive symptoms in 2016. We evaluated model fit using the IFI, CFI, AGFI, and NFI indices, which indicate excellent fit when they exceed 0.90 (43, 44). A RMSEA value of <0.05 indicated a close fit (45, 46).

## 3. Results

### 3.1. Descriptive analysis

Sample characteristics and descriptive statistics were presented in Table 1. The final sample included 2,105 (51.34%) males and 1995 (48.66%) females. The mean age of respondents was 34.35 years (SD = 9.56). 2,338 (57.02%) participants’ education was high school or less, and 1762 (42.98%) participants had a college education. 2,703 (65.93%) participants were married, and 1,397 (34.07%) participants were single. The mean score for depressive symptoms of the respondents was 0.58 (SD = 0.41) in 2016, and the score increased to 0.85 (SD = 0.45) in 2020.

**Table 1:** Participant characteristics and descriptive statistics (*n* = 4,100).

Variables	*N* (%)	Mean (SD)
Age(years)		34.35 (9.56)
Gender		
Male	2,105 (51.34%)	
Female	1995 (48.66%)	
Education		
High school or less	2,338 (57.02%)	
College or more	1762 (42.98%)	
Marital status		
Married/living with partner	2,703 (65.93%)	
Single, never been married	1,397 (34.07%)	
PII2016		3.53 (0.79)
FIU2018		5.11 (1.26)
SSS2018		3.28 (0.61)
CESD2016		0.58 (0.41)
CESD2020		0.85 (0.45)

PII2016, perceived importance of the internet in 2016; FIU2018, The frequency of internet use in 2018; SSS2018, Subjective socioeconomic status in 2018; CESD2020, Self-rated depressive symptoms in 2016; CESD2020, Self-rated depressive symptoms in 2020.

Correlations between all variables can be seen in Table 2. PII2016 (*r* = −0.033, *p* < 0.001), FIU2018 (*r* = −0.031, *p* < 0.05), and SSS2018 (*r* = −0.177, *p* < 0.001) were negatively correlated with CESD2016; FIU2018 (*r* = −0.044, *p* < 0.01), and SSS2018 (*r* = −0.182, *p* < 0.001) were negatively correlated with CESD2020; CESD2016 (*r* = 0.360, *p* < 0.001) were positively correlated with CESD2020. Age was negatively correlated with PII2016 (*r* = −0.065, *p* < 0.001) and FIU2018 (*r* = −0.107, *p* < 0.001). Besides, Gender, Education, and Marital status were significantly associated with other independent or dependent variables in different degrees.

**Table 2:** Correlations or covariances between all used variables (*n* = 4,100).

	2	3	4	5	6	7	8	9. Marital status
1. PII2016	0.273^***^	0.068^***^	−0.033^*^	0.005	−0.065^***^	2.052^*^	−8.999^***^	1.279
2. FIU2018		0.052^**^	−0.031^*^	−0.044^**^	−0.107^***^	−3.505^***^	−26.745^***^	6.764^***^
3. SSS2018			−0.177^***^	−0.182^***^	0.014	−1.833	−1.590	−2.759^**^
4. CESD2016				0.360^***^	0.012	1.554	5.370^***^	0.160
5. CESD2020					0.007	2.958^**^	−1.002^***^	−5.347^***^
6. Age						−2.445^*^	10.230^***^	−38.621^***^
7. Gender							3.205	27.290^***^
8. Education								124.499^***^

Significance level: ^*^0.05; ^**^0.01; ^***^0.001. PII2016, perceived importance of the internet in 2016; FIU2018, The frequency of internet use in 2018; SSS2018, Subjective socioeconomic status in 2018; CESD2020, Self-rated depressive symptoms in 2016; CESD2020, Self-rated depressive symptoms in 2020.

### 3.2. Structural equation modeling results

**Figure 3:**
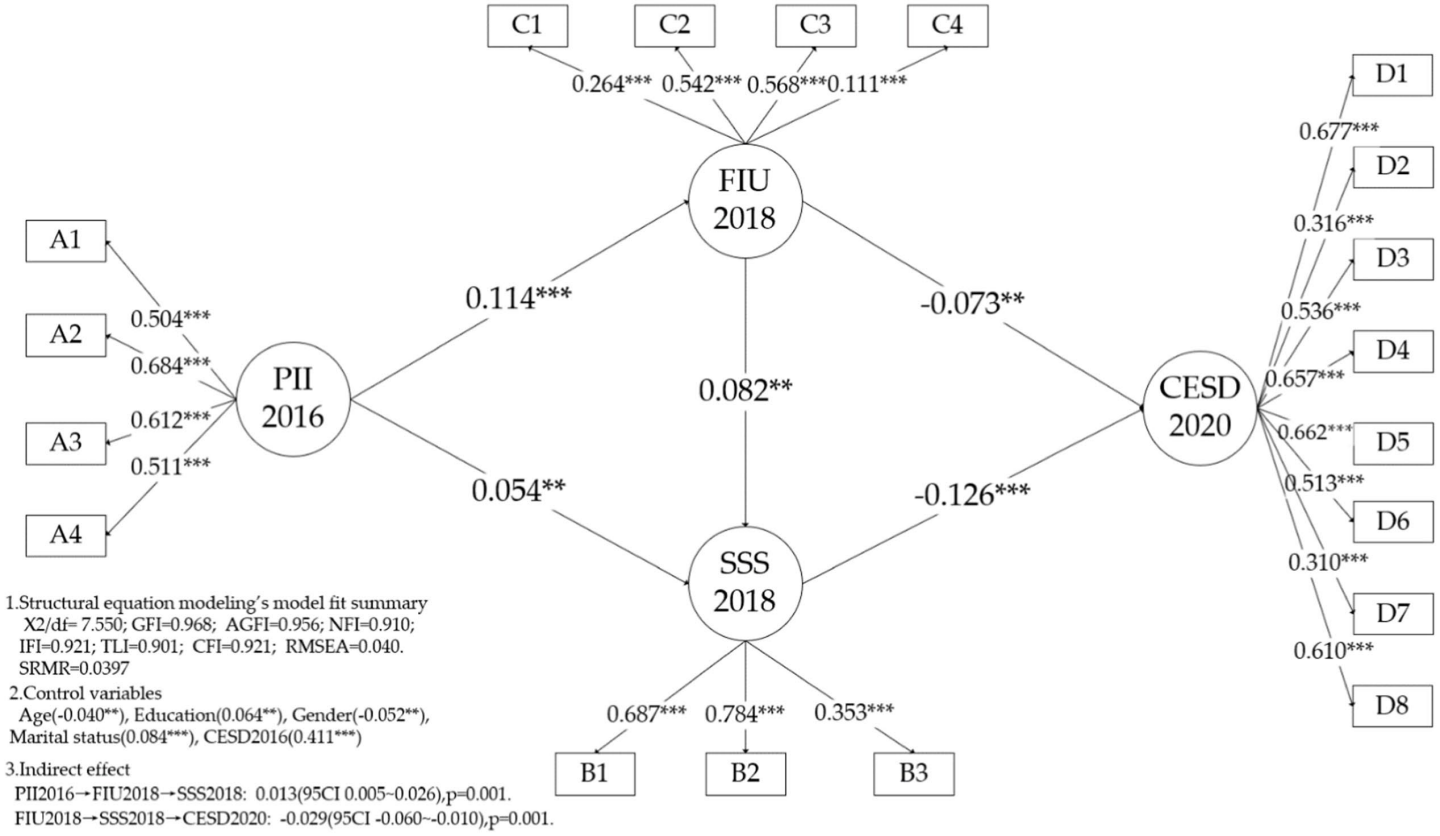
Structural equation modeling results. PII2016, perceived importance of the internet in 2016; FIU2018, The frequency of internet use in 2018; SSS2018, Subjective socioeconomic status in 2018; CESD2020, Self-rated depressive symptoms in 2020.

The model fit indices showed that the hypothesized model fit the data well. Specifically, the GFI (0.968), NFI (0.910), IFI (0.921), TLI (0.901), CFI (0.921), and AGFI (0.955) values were above the reference point (0.9), the RMSEA (0.040) and SRMR (0.0397) values were lower than that of the reference point (0.05). The goodness-of-fit indices for the model are shown in Figure 3.

The results of the study indicate that the perceived importance of the Internet in 2016 was positively associated with the frequency of Internet use (*β* = 0.114, *p* < 0.001) and subjective socioeconomic status (*β* = 0.054, *p* < 0.01) in 2018, thus supporting hypothesis H1. Additionally, the frequency of Internet use (*β* = −0.073, *p* < 0.01) and subjective socioeconomic status (*β* = −0.126, *p* < 0.001) in 2018 were negatively associated with depressive symptoms in 2020, which supports hypothesis H2. Notably, the mediating effect of the frequency of Internet use on the relationship between perceived importance of the Internet and subjective socioeconomic status was fully supported. Similarly, the mediating effect of subjective socioeconomic status on the relationship between the frequency of Internet use and depressive symptoms was also fully supported. Specifically, the frequency of Internet use and subjective socioeconomic status had a mediating effect of 0.013 (95%CI 0.005, 0.026) and-0.029 (95%CI-0.060, −0.010), respectively.

Furthermore, the study found a significant association between depressive symptoms in 2016 and 2020 (*β* = 0.411, *p* < 0.001), with age and gender negatively influencing CESD2020 (*β*_age_ = −0.040, *p* < 0.01; *β*_gender_ = −0.052, *p* < 0.01) and education and marital status positively associated with CESD2020 (*β*_education_ = 0.064, *p* < 0.01; *β*_marital status_ = 0.084, *p* < 0.001). These findings suggest that younger, female, unmarried, and well-educated individuals may experience higher levels of depressive symptoms.

## 4. Discussion

The primary objective of this study was to examine the impact of individuals’ cognitions about the Internet on their depressive symptoms. Overall, the study findings support our hypotheses.

The study results indicate a positive relationship between the perceived importance of the internet, the frequency of internet use, and subjective socioeconomic status. This conclusion aligns with prior research on the topic. Previous studies have demonstrated that the perceived importance of the internet is a significant factor in determining its frequency of use. For example, Lin and Lu (47) found that individuals who perceive the internet as more important use it more frequently than those who do not. This suggests that an individual’s cognition influences their behavior, with cognition being a basic mental process that shapes an individual’s decisions, behavior, and ability to assess and avoid external threats. Thus, individuals who place a high value on the internet are more likely to use it to improve their lives, as per the cognitive theoretical model. Moreover, the study revealed that the perceived importance of the internet’s effect on subjective socioeconomic status is mediated by the frequency of internet use. This relationship could be explained by the fact that internet use provides individuals with access to resources and information, which influences their perception of their social and economic position relative to others in society. Additionally, frequent internet use can bridge digital inequalities, promote health information-seeking, reduce health disparities, and enhance well-being, as demonstrated by previous studies (48–51). However, it is essential to note that this relationship may not be causal. For instance, studies indicate that individuals with higher subjective socioeconomic status tend to use the internet more frequently than those with lower subjective socioeconomic status (52), indicating that subjective socioeconomic status may influence an individual’s frequency of internet use. Overall, the findings suggest that policies aimed at expanding internet access should also focus on individuals’ perceptions of its importance. Policymakers could consider strategies to raise awareness of the internet and digital technologies’ benefits, particularly among individuals who do not currently value its importance.

Secondly, the study’s results reveal a negative correlation between depressive symptoms and both the frequency of internet use and subjective socioeconomic status. This finding is consistent with previous research, which has demonstrated that the internet has numerous beneficial effects on mental health (53, 54), such as increasing life satisfaction and reducing negative emotions (55, 56). Studies have also shown that internet usage can expand social networks, enhance social participation, and improve social capital, all of which have been linked to lower levels of depression (57–61). Similarly, individuals with a higher subjective socioeconomic status are less likely to experience depressive symptoms due to their better access to resources and greater sense of control over their circumstances (62, 63). This finding is supported by research that indicates that high subjective socioeconomic status has a protective effect against depression and positively affects psychological factors such as well-being, self-esteem, and perceived stress (64–66). Overall, the negative associations between the frequency of internet use and subjective socioeconomic status with depressive symptoms highlight the potential benefits of addressing inequalities in internet access and use, as well as addressing social determinants of mental health such as socioeconomic status. The finding also highlights the importance of addressing socioeconomic inequality in access to the internet and digital technologies. Policymakers could implement measures aimed at reducing the digital divide, such as providing subsidies for internet access and digital devices for low-income households and investing in infrastructure to expand access to high-speed internet in rural and remote areas.

Finally, the finding that the earlier depressive symptoms revealed a significant effect on later depressive symptoms, which is consistent with past studies (67, 68). Depression is a negative psychological state that can easily reoccur (60, 61, 69). Studies have shown that mild depression may get recover without treatment, but moderate and severe depression without timely intervention may cause long-term symptoms and even suicidal tendencies (68). Therefore, we advocate taking intervention measures for these people who are fall into earlier depressive symptoms, helping them relieve these depressive symptoms. Moreover, the current found indicating that young, well-educated, female, unmarried people may be at higher risk of having depressive symptoms. While these findings may seem counterintuitive, they are supported by previous research that has identified similar risk factors for depression. For example, studies have consistently shown that women are more likely than men to experience depression (70), and that individuals with higher levels of education may also be at increased risk (71). The fact that young, unmarried individuals are also at increased risk for depression is also consistent with previous research (72, 73). This may be because young adulthood is a time of significant life transitions and stressors, such as starting a new job or relationship, and because the lack of social support that can come with being unmarried can exacerbate these stressors. These findings have important implications for mental health professionals, policymakers, and public health practitioners. Mental health professionals need to be aware of the elevated risk for depression in these populations and tailor their interventions accordingly. They should also consider specific risk factors that may be more prevalent in these groups, such as academic or career-related stress, social isolation, and relationship issues.

## 5. Limitations

Despite the contributions of the current study, it also has some limitations.

Firstly, one limitation of the present study is the possibility that some correlations reported in Table 2 may have been influenced by a large sample size, leading to statistical significance despite weak effect sizes. It is important to note that statistically significant findings may not necessarily translate to practical significance. Weak correlations do not always imply a strong or meaningful relationship between variables, and as such, their interpretation must be approached with caution. To address this limitation, future research may consider using different statistical techniques, increasing the sample size, or collecting additional data to better understand the nature of the relationship between the variables. Additionally, acknowledging this limitation is essential when interpreting the results of this study, as it highlights the need for further investigation into the relationship between the variables of interest.

Secondly, the present study is related to the sample size and representativeness of the data. Despite the use of nationally representative data from the CFPS, the current sample size was relatively small compared to the original baseline data. Moreover, certain questionnaires were only administered to a subsample, resulting in a significant amount of missing data. As such, the present findings may lack generalizability to other populations or contexts. To address this limitation, future research could use larger and more diverse samples, as well as more comprehensive measures of the variables of interest. Additionally, it may be useful to investigate other behaviors and emotions that may alleviate depressive symptoms and promote better emotional health outcomes, using the CFPS data as delimiting variables. By doing so, a more nuanced understanding of the relationship between these factors can be gained, and interventions can be developed to improve emotional well-being in various contexts.

Thirdly, the present study relates to the measurement of subjective socioeconomic status, which was assessed using only three items. While three-item scales have been acknowledged in the literature as valuable instruments for measuring SES, their validity and reliability may be further improved by utilizing multi-item scales in future research. Furthermore, to enhance the validity of the findings in the present study, it may be beneficial for future research to employ more comprehensive scales to measure the perceived importance of the Internet, as well as the frequency of Internet use. Doing so would allow for a more detailed understanding of the relationship between these variables and depressive symptoms, and may uncover potential mechanisms underlying these associations. By addressing these limitations, future studies can better inform interventions aimed at improving emotional well-being in different populations and settings.

A fourth limitation of the present study pertains to the analysis of longitudinal data. Although an implied model was used in the present study, a cross-lagged model would be the optimal choice for analyzing longitudinal data, as it takes into account the values of all previous variables in the next one. Unfortunately, the CFPS data did not meet the requirements for this model. To overcome this limitation, future research may consider using a cross-lagged model to analyze research hypotheses, thereby providing a more nuanced understanding of the relationship between variables over time. Such an approach can be useful in identifying potential causal pathways.

Finally, a limitation of the present study is that the measures used did not explore the personal value of the Internet or the emotional states experienced when using it. Rather, the study solely measured the perceived importance of the Internet to the participants. It is important to note that a person may perceive the Internet as highly important to themselves but may still engage in problematic Internet use, which can ultimately lead to poor social functioning and emotional distress. Such a pathway has been well-documented in the literature. Therefore, the results of the present study may require further analysis to fully understand the underlying mechanisms of the observed associations between the variables of interest. Future research may consider employing more nuanced measures that capture the multidimensional nature of Internet use and its effects on emotional well-being, including measures of emotional experiences and problematic Internet use.

## 6. Conclusion

In conclusion, our study demonstrates that the perceived importance of the internet has a protective effect on individuals’ depressive symptoms. The conclusion proves the notion of cognitive theory of emotion with data, which states individual’s perception of things can affect they feelings and behaviors, this also implies that the perception of the Internet also affects one’s mood, not only the behavior of Internet use. In addition, these findings have positive implications for public health and public policy. From a public health perspective, these findings suggest that the internet may have a critical role in promoting mental health and preventing depression. Given the significant burden that depression imposes on individuals and society, identifying protective factors that can help mitigate its risk is of paramount importance. Therefore, interventions aimed at promoting the use of the internet and enhancing individuals’ perceptions of its importance may be beneficial in preventing depression. Moreover, this study underscores the necessity of promoting equitable access to the internet, particularly in disadvantaged communities. The internet has the potential to serve as a valuable resource for individuals facing social and economic challenges, providing access to information, resources, and social support that may help mitigate the risk of depression. From a policy perspective, these findings suggest that policymakers should consider the potential mental health benefits of promoting internet access and use. Policies aimed at increasing internet access, affordability, and digital literacy may help reduce disparities in mental health outcomes and improve overall population mental health. In sum, the present study’s findings suggest that the perceived importance of the internet may play a protective role in preventing depression, and promoting equitable access to the internet has the potential to improve population mental health. Therefore, further research in this area is warranted to better understand the mechanisms underlying the relationship between internet use and mental health, and to inform the development of evidence-based interventions aimed at improving mental health outcomes.

## Data availability statement

The original contributions presented in the study are included in the article/supplementary material, further inquiries can be directed to the corresponding authors.

## Ethics statement

The studies involving human participants were reviewed and approved by the Biomedical Ethics Review Committee of Peking University approved this study (IRB00001052-14010), and all participants signed informed consent. The patients/participants provided their written informed consent to participate in this study.

## Author contributions

ZW and JbZ: conceptualization. JwZ and Y-WX: funding acquisition. ZW: methodology, software, and writing–original draft. JbZ, JwZ, and MJ: validation. MJ: visualization. JbZ, MJ, JwZ, and Y-WX: writing–review and editing. All authors will be informed about each step of manuscript processing including submission, revision, revision reminder, etc. via emails from our system or assigned Assistant Editor.

## Funding

This research was funded by the National Natural Science Foundation of China [no. 72274023]. The National Natural Science Foundation of China was an institution directly under the jurisdiction of the State Council, tasked with the administration of the National Natural Science Fund from the Central Government.

## Conflict of interest

The authors declare that the research was conducted in the absence of any commercial or financial relationships that could be construed as a potential conflict of interest.

## Publisher’s note

All claims expressed in this article are solely those of the authors and do not necessarily represent those of their affiliated organizations, or those of the publisher, the editors and the reviewers. Any product that may be evaluated in this article, or claim that may be made by its manufacturer, is not guaranteed or endorsed by the publisher.

## Abbreviations

CFPS: The China Family Panel Studies
PII2016: perceived importance of the internet in 2016
FIU2018: The frequency of internet use in 2018
SSS2018: Subjective socioeconomic status in 2018
CESD2016: Self-rated depressive symptoms in 2016
CESD2020: Self-rated depressive symptoms in 2020

## References

[1] International Telecommunication Union. *Ageing in a digital world: from vulnerable to valuable*. International Telecommunication Union, (2022). 10–11.

[2] Yuan H. Internet use and mental health problems among older people in Shanghai, China: the moderating roles of chronic diseases and household income. Aging Ment Health. (2021) 25:657–63. doi: 10.1080/13607863.2020.1711858 31928208

[3] Ueno T Ito K Murai T Fujiwara H. Mental health problems and their association with internet use in medical residents. Front Public Health. (2020) 8:1–8. doi: 10.3389/fpubh.2020.587390 33194994 PMC7641600

[4] Žaja N Vukojević J Žarko T Marelić M Vidović D Rukavina TV. Internet use among patients with schizophrenia and depression. Int J Environ Res Public Health. (2022) 19:1–13. doi: 10.3390/ijerph19095695 PMC9104824 35565091

[5] Yen JY Ko CH Yen CF Wu HY Yang MJ. The comorbid psychiatric symptoms of internet addiction: attention deficit and hyperactivity disorder (ADHD), depression, social phobia, and hostility. J Adolesc Health. (2007) 41:93–8. doi: 10.1016/j.jadohealth.2007.02.002 17577539

[6] Hamilton MA. Rating scale for depression. J Neurol Neurosurg Psychiatry. (1960) 23:56–62. doi: 10.1136/jnnp.23.1.56 14399272 PMC495331

[7] World Health Organization. Depression and other common mental disorders. Geneva: World Health Organization (2017). 7–9.

[8] Lane NE Hoben M Amuah JE Hogan DB Baumbusch J Gruneir A. Prevalence and correlates of anxiety and depression in caregivers to assisted living residents during COVID-19: a cross-sectional study. BMC Geriatr. (2022) 22:1–12. doi: 10.1186/s12877-022-03294-y 35962356 PMC9372518

[9] Folkman S Lazarus RS. If it changes it must be a process: study of emotion and coping during three stages of a college examination. Journal of personality and social psychology. (1985) 48:150. doi: 10.1037/0022-3514.48.1.150 2980281

[10] Lazarus RS. Emotion and adaptation. (1991) Oxford University Press.

[11] Diotaiuti P Mancone S Corrado S De Risio A Cavicchiolo E Girelli L. Internet addiction in young adults: the role of impulsivity and codependency. Front Psych. (2022) 13:893861. doi: 10.3389/fpsyt.2022.893861 PMC9485605 36147985

[12] Diotaiuti P Girelli L Mancone S Corrado S Valente G Cavicchiolo E. Impulsivity and depressive brooding in internet addiction: a study with a sample of Italian adolescents during COVID-19 lockdown. Front Psych. (2022) 13:941313. doi: 10.3389/fpsyt.2022.941313 PMC9309336 35898621

[13] Anderson EL Steen E Stavropoulos V. Internet use and problematic internet use: a systematic review of longitudinal research trends in adolescence and emergent adulthood. Int J Adolesc Youth. (2017) 22:430–54. doi: 10.1080/02673843.2016.1227716

[14] Yulong G Kalibatseva Z Song X. Effective use of online depression information and associated literacies among US college students. Health Promot Int. (2021) 36:1020–8. doi: 10.1093/heapro/daaa116 33277908

[15] Katikalapudi R Chellappan S Montgomery F Wunsch D Lutzen K. Associating internet usage with depressive behavior among college students. IEEE Technol Soc Mag. (2012) 31:73–80. doi: 10.1109/MTS.2012.2225462

[16] Hwang JM Cheong PH Feeley TH. Being young and feeling blue in Taiwan: examining adolescent depressive mood and online and offline activities. New Media Soc. (2009) 11:1101–21. doi: 10.1177/1461444809341699

[17] Casale S Lecchi S Fioravanti G. The association between psychological well-being and problematic use of internet communicative services among Young people. J Psychol. (2015) 149:480–97. doi: 10.1080/00223980.2014.905432 25975575

[18] Mairs K McNeil H McLeod J Prorok JC Stolee P. Online strategies to facilitate health-related knowledge transfer: a systematic search and review. Health Inf Libr J. (2013) 30:261–77. doi: 10.1111/hir.12048 24251889

[19] Cohall AT Nye A Moon-Howard J Kukafka R Northridge ME. Computer use, internet access, and online health searching among Harlem adults. Am J Health Promot. (2016) 25:325–33. doi: 10.4278/ajhp.090325-QUAN-121 21534835

[20] Bauernschuster S Falck O Woessmann L. Surfing alone? The internet and social capital: evidence from an unforeseeable technological mistake. J Public Econ. (2014) 117:73–89. doi: 10.1016/j.jpubeco.2014.05.007

[21] Cotten SR Ford G Ford S Hale TM. Internet use and depression among older adults. Comput Hum Behav. (2012) 28:496–9. doi: 10.1016/j.chb.2011.10.021

[22] Mitchell ME Lebow JR Uribe R Grathouse H Shoger W. Internet use, happiness, social support and introversion: a more fine-grained analysis of person variables and internet activity. Comput Hum Behav. (2011) 27:1857–61. doi: 10.1016/j.chb.2011.04.008

[23] Zhan G Zhou Z. Mobile internet and consumer happiness: the role of risk. Internet Res. (2018) 28:785–803. doi: 10.1108/IntR-11-2016-0340

[24] Wartberg L Brunner R Kriston L Durkee T Parzer P Fischer-Waldschmidt G. Psychopathological factors associated with problematic alcohol and problematic internet use in a sample of adolescents in Germany. Psychiatry Res. (2016) 240:272–7. doi: 10.1016/j.psychres.2016.04.057 27138817

[25] Bozoglan B Demirer V Sahin I. Problematic internet use: functions of use, cognitive absorption, and depression. Comput Hum Behav. (2014) 37:117–23. doi: 10.1016/j.chb.2014.04.042

[26] Park S Hong K-EM Park EJ Ha KS Yoo HJ. The association between problematic internet use and depression, suicidal ideation and bipolar disorder symptoms in Korean adolescents. Aust N Z J Psychiatry. (2013) 47:153–9. doi: 10.1177/0004867412463613 23047959

[27] Kokka I Mourikis I Nicolaides NC Darviri C Bacopoulou F. Exploring the effects of problematic internet use on adolescent sleep: a systematic review. Int J Environ Res Public Health. (2021) 18:1–14. doi: 10.3390/ijerph18020760 PMC7830369 33477410

[28] Odaci H Celik CB. Does internet dependence affect young people's psycho-social status? Intrafamilial and social relations, impulse control, coping ability and body image. Comput Hum Behav. (2016) 57:343–7. doi: 10.1016/j.chb.2015.12.057

[29] Young S. Internet addiction: the emergence of a new clinical disorder. Cyberpsychol Behav. (2009) 1:237–44. doi: 10.1089/cpb.1998.1.237

[30] Ko C-H Liu T-L Wang P-W Chen C-S Yen C-F Yen J-Y. The exacerbation of depression, hostility, and social anxiety in the course of internet addiction among adolescents: a prospective study. Compr Psychiatry. (2014) 55:1377–84. doi: 10.1016/j.comppsych.2014.05.003 24939704

[31] Suhail K Bargees Z. Effects of excessive internet use on undergraduate students in Pakistan. Cyberpsychol Behav. (2006) 9:297–307. doi: 10.1089/cpb.2006.9.297 16780397

[32] Demakakos P Nazroo J Breeze E Marmot M. Socioeconomic status and health: the role of subjective social status. Soc Sci Med. (2008) 67:330–40. doi: 10.1016/j.socscimed.2008.03.038 18440111 PMC2547480

[33] Singh-Manoux A Adler NE Marmot MG. Subjective social status: its determinants and its association with measures of ill-health in the Whitehall II study. Soc Sci Med. (2003) 56:1321–33. doi: 10.1016/S0277-9536(02)00131-4 12600368

[34] Singh-Manoux A Marmot MG Adler NE. Does subjective social status predict health and change in health status better than objective status? Psychosom Med. (2005) 67:855–61. doi: 10.1097/01.psy.0000188434.52941.a0 16314589

[35] Operario D Adler NE Williams DR. Subjective social status: reliability and predictive utility for global health. Psychol Health. (2004) 19:237–46. doi: 10.1080/08870440310001638098

[36] Lundberg J Kristenson M. Is subjective status influenced by psychosocial factors? Soc Indic Res. (2008) 89:375–90. doi: 10.1007/s11205-008-9238-3

[37] Callan MJ Kim H Matthews WJ. Predicting self-rated mental and physical health: the contributions of subjective socioeconomic status and personal relative deprivation. Front Psychol. (2015) 6:1–14. doi: 10.3389/fpsyg.2015.01415 26441786 PMC4585190

[38] Assari S Preiser B Lankarani MM Caldwell CH. Subjective socioeconomic status moderates the association between discrimination and depression in African American youth. Brain Sci. (2018) 8:1–14. doi: 10.3390/brainsci8040071 PMC5924407 29677115

[39] Cotten SR Ford G Ford S Hale TM. Internet use and depression among retired older adults in the United States: a longitudinal analysis. J Gerontol. (2014) 69:763–71. doi: 10.1093/geronb/gbu018 24671896

[40] Zhang H Wang H Yan H Wang X. Impact of internet use on mental health among elderly individuals: a difference-in-differences study based on 2016–2018 CFPS data. Int J Environ Res Public Health. (2022) 19:1–13. doi: 10.3390/ijerph19010101 PMC8749999 35010361

[41] Radloff LS. The ces-d scale a self-report depression scale for research in the general population. Appl Psychol Meas. (1977) 1:385–401.

[42] Zhang Y Li Y Li WH Jiang L Tao Q. The reliability and validity of the center for epidemiologic studies depression scale (CES-D) for Chinese university students. Front Psych. (2019) 10:1–12. doi: 10.3389/fpsyt.2019.00315 PMC6537885 31178764

[43] Bentler PM. Comparative fit indexes in structural models. Psychol Bull. (1990) 107:238–46. doi: 10.1037/0033-2909.107.2.238 2320703

[44] Hu LT Bentler PM. Cutoff criteria for fit indexes in covariance structure analysis: conventional criteria versus new alternatives. Struct Equal Model. (1999) 6:1–55. doi: 10.1080/10705519909540118

[45] Browne MW Cudeck R. Alternative ways of assessing model fit. Sociol Methods Res. (1992) 21:230–58. doi: 10.1177/0049124192021002005

[46] Steiger JH. Structural model evaluation and modification: an interval estimation approach. Multivar Behav Res. (1990) 25:173–80. doi: 10.1207/s15327906mbr2502_4 26794479

[47] Lin CA Lu HP. Why people use social networking sites: an empirical study integrating network externalities and motivation theory. Comput Hum Behav. (2011) 27:1152–61. doi: 10.1016/j.chb.2010.12.009

[48] Wang MP Wang X Lam TH Viswanath K Chan SS. Health information seeking partially mediated the association between socioeconomic status and self-rated health among Hong Kong Chinese. PLoS One. (2013) 8:e82720. doi: 10.1371/journal.pone.0082720 24349347 PMC3862642

[49] Mu A Deng Z Wu X Zhou L. Does digital technology reduce health disparity? Investigating difference of depression stemming from socioeconomic status among Chinese older adults. BMC Geriatr. (2021) 21:1–14. doi: 10.1186/s12877-021-02175-0 33882865 PMC8059190

[50] Perez SL Kravitz RL Bell RA Chan MS Paterniti DA. Characterizing internet health information seeking strategies by socioeconomic status: a mixed methods approach. BMC Med Inform Decis Mak. (2016) 16:1–8. doi: 10.1186/s12911-016-0344-x 27506607 PMC4979125

[51] Das S Chatterjee A. Impacts of ICT and digital finance on poverty and income inequality: a sub-national study from India. Inf Technol Dev. (2023) 2023:1–28. doi: 10.1080/02681102.2021.1962234

[52] Wangberg SC Andreassen HK Prokosch H-U Santana SMV Sørensen T Chronaki CE. Relations between internet use, socio-economic status (SES), social support and subjective health. Health Promot Int. (2008) 23:70–7. doi: 10.1093/heapro/dam039 18083686

[53] Estacio EV Whittle R Protheroe J. The digital divide: examining socio-demographic factors associated with health literacy, access and use of internet to seek health information. J Health Psychol. (2019) 24:1668–75. doi: 10.1177/1359105317695429 28810415

[54] Jiang S Beaudoin CE. Health literacy and the internet: an exploratory study on the 2013 HINTS survey. Comput Hum Behav. (2016) 58:240–8. doi: 10.1016/j.chb.2016.01.007

[55] Lam SSM Jivraj S Scholes S. Exploring the relationship between internet use and mental health among older adults in England: longitudinal observational study. J Med Internet Res. (2020) 22:1–10. doi: 10.2196/15683 PMC7420689 32718913

[56] Liu Q Pan H Wu Y. Migration status. Internet use, and social participation among middle-aged and older adults in China: consequences for depression. Int J Environ Res Public Health. (2020) 17:1–13. doi: 10.3390/ijerph17166007 PMC7459605 32824867

[57] Cheng H Furnham A. Personality, self-esteem, and demographic predictions of happiness and depression. Personal Individ Differ. (2003) 34:921–42. doi: 10.1016/S0191-8869(02)00078-8

[58] Shi X He X Pan D Qiao H Li J. Happiness, depression, physical activity and cognition among the middle and old-aged population in China: a conditional process analysis. PeerJ. (2022) 10:1–18. doi: 10.7717/peerj.13673 PMC9248807 35782096

[59] Seo EH Kim SG Kim SH Kim JH Park JH Yoon HJ. Life satisfaction and happiness associated with depressive symptoms among university students: a cross-sectional study in Korea. Ann General Psychiatry. (2018) 17:1–9. doi: 10.1186/s12991-018-0223-1 PMC6297950 30568720

[60] Yang SN Chueh C-H Peng L-N Tsai Y-W. Impacts of intervals between sequential development of depression and dementia in older adults: a nationwide population-based study. Arch Gerontol Geriatr. (2022) 101:104693–5. doi: 10.1016/j.archger.2022.104693, PMID: 35390572

[61] Yang HL Zhang S Cheng S. A study on the impact of internet use on depression among Chinese older people under the perspective of social participation. BMC Geriatr. (2022) 22:1–11. doi: 10.1186/s12877-022-03359-y 35999498 PMC9398499

[62] Dolbier CL Rush TE Sahadeo LS Shaffer ML Thorp J Community Child Health Network Investigators. Relationships of race and socioeconomic status to postpartum depressive symptoms in rural African American and non-Hispanic white women. Matern Child Health J. (2013) 17:1277–87. doi: 10.1007/s10995-012-1123-7 22961387 PMC3584227

[63] Zou H Chen Y Fang W Zhang Y Fan X. The mediation effect of health literacy between subjective social status and depressive symptoms in patients with heart failure. J Psychosom Res. (2016) 91:33–9. doi: 10.1016/j.jpsychores.2016.10.006 27894460

[64] Huang S Hou J Sun L Dou D Liu X Zhang H. The effects of objective and subjective socioeconomic status on subjective well-being among rural-to-urban migrants in China: the moderating role of subjective social mobility. Front Psychol. (2017) 8:1–9. doi: 10.3389/fpsyg.2017.00819 28588531 PMC5439243

[65] Cundiff JM Matthews KA. Is subjective social status a unique correlate of physical health? A meta-analysis. Health Psychol. (2017) 36:1109–25. doi: 10.1037/hea0000534 28726474 PMC5709157

[66] You J Zhu Y Liu S Wang C Wang P Du H. Socioeconomic disparities in psychological health: testing the reserve capacity model in a population-based sample of Chinese migrants. J Health Psychol. (2021) 26:1538–48. doi: 10.1177/1359105319882763 31621415

[67] DiMatteo MR Lepper HS Croghan TW. Depression is a risk factor for noncompliance with medical treatment: Meta-analysis of the effects of anxiety and depression on patient adherence. Arch Intern Med. (2020) 160:2101–7. doi: 10.1001/archinte.160.14.2101 10904452

[68] Kessler RC Berglund P Demler O. The epidemiology of major depressive disorder: results from the National Comorbidity Survey Replication (NCS-R). JAMA. (2003) 289:3095–105. doi: 10.1001/jama.289.23.3095 12813115

[69] Ismail Z Corinne Fischer W McCall V. What characterizes late-life depression? Psychiatr Clin N Am. (2013) 36:483–96. doi: 10.1016/j.psc.2013.08.010 24229652

[70] Alonso J Angermeyer MC Bernert S Bruffaerts R Brugha TS Bryson H. Prevalence of mental disorders in Europe: results from the European study of the epidemiology of mental disorders (ESEMeD) project. Acta Psychiatr Scand. (2004) 109:21–7. doi: 10.1111/j.1600-0047.2004.00327.x 15128384

[71] Alloy LB Abramson LY Smith JM Gibb BE Neeren AM. Role of parenting and maltreatment histories in unipolar and bipolar mood disorders: mediation by cognitive vulnerability to depression. Clin Child Fam Psychol Rev. (2006) 9:23–64. doi: 10.1007/s10567-006-0002-1 16718583

[72] Kessler RC Berglund P Demler O Jin R Merikangas KR Walters EE. Lifetime prevalence and age-of-onset distributions of DSM-IV disorders in the National Comorbidity Survey Replication. Arch Gen Psychiatry. (2005) 62:593–602. doi: 10.1001/archpsyc.62.6.593 15939837

[73] Lorant V Deliège D Eaton W Robert A Philippot P Ansseau M. Socioeconomic inequalities in depression: a meta-analysis. Am J Epidemiol. (2003) 157:98–112. doi: 10.1093/aje/kwf182 12522017

